# Adsorptive Gas Sensor Response Forecasting to Enable Breath-by-Breath Analysis

**DOI:** 10.3390/s26072234

**Published:** 2026-04-04

**Authors:** Samuel Bellaire, Samir Rawashdeh, Kirby P. Mayer, Jamie L. Sturgill

**Affiliations:** 1 College of Engineering & Computer Science, University of Michigan-Dearborn, Dearborn, MI 48128, USA; 2 Department of Physical Therapy, College of Health Sciences, University of Kentucky, Lexington, KY 40506, USA; kpmaye2@uky.edu; 3 Department of Microbiology, Immunology, and Molecular Genetics, College of Medicine, University of Kentucky, Lexington, KY 40506, USA; jamie.sturgill@uky.edu

**Keywords:** gas sensors, MOS gas sensors, E-nose, electronic nose, forecasting

## Abstract

MOS gas sensors have proven to be useful in electronic noses, which utilize these sensors to detect volatile organic compounds in human breath to detect various lung diseases. Unfortunately, the long settling time of MOS gas sensors is ill-suited to measuring human breath, where complete breathing cycles are often shorter than 5 s. Existing studies circumvent this limitation by collecting gas samples and injecting them into a sealed chamber to react with the sensors. However, it would be convenient if breath-by-breath analysis could be conducted without the need to store breath samples. To accomplish this, we present a novel forecasting methodology to predict the final value $t_{\infty }$ of a gas sensor’s response based on its initial transient behavior. To do this, we present and validate a second-order mathematical model of the sensors’ response characteristics, which we then use in our preliminary work using neural networks to predict the final sensor value. Although some challenges were encountered, the initial results are encouraging, and we plan to extend our study in the future to collect a more expansive dataset and explore the use of other types of machine learning algorithms for this application.

## 1. Introduction

### 1.1. Motivation

Chronic obstructive pulmonary diseases (COPD) are a family of diseases, such as chronic bronchitis and emphysema, that permanently inhibit an individual’s pulmonary function. COPD accounted for 3.23 million deaths in 2019 according to the World Health Organization [1], and is among the most common diseases in the world, with 6.4% of Americans having been diagnosed with COPD in 2018 [2].

Individuals with COPD often exhibit symptoms that result in impairment of their physical abilities due to their decreased lung function, which drastically reduces their quality of life. Exacerbation of these symptoms frequently necessitates readmission to the hospital [3,4,5], leading to increased healthcare utilization. It has also been found that patients with COPD exhibit an elevated risk for other respiratory complications, such as influenza, pneumonia, or lung cancers [1].

The risks associated with COPD make early diagnosis and treatment imperative. There are a number of different methods that have been developed to assist in the diagnosis of COPD; perhaps the most prevalent is the pulmonary function test (PFT). PFTs typically involve spirometry, which measures the forced expiratory volume (FEV) and forced vital capacity (FVC) of a patient’s lungs, among other measures [6,7]. More recently, researchers have adapted so-called “electronic noses” to assist in the diagnosis of lung diseases, which are briefly outlined below in Section 1.2.

### 1.2. Related Work

Electronic noses, or E-noses, are devices that employ a suite of electronic sensors to detect the presence of various gases. They have been used in a number of medical applications, such as the Lab-on-Mask [8] by Pan et al. and FaceBit [9] by Curtiss et al. These “smart masks” were designed to enable remote respiratory monitoring during the COVID-19 pandemic, among other functions. Myers et al. developed the OOCOO smart mask, which is designed to allow pre-operative risk evaluation of lung cancer patients without the use of specialized equipment, which may not always be available for use [10]. In non-medical applications, researchers have also looked at E-noses for environmental monitoring, such as evaluation of tobacco smoke [11] and air quality monitoring in waste water treatment plants [12]. In another paper, Suresh et al. review the use of E-nose systems for various agricultural contexts in the literature, such as the evaluation of seed stockpiles for seed quality and contaminants [13].

E-noses have also been used in the literature to detect volatile organic compounds (VOCs). Researchers have shown that hydrocarbons, alcohols, and aldehydes in exhaled breath are biomarkers of lung diseases [14,15,16]. Recent works in the literature have demonstrated advances in MOS-based sensing for high-sensitivity detection of acetone [17,18], which is particularly useful for non-invasive detection of diabetes.

A number of recent studies have utilized E-noses to detect these VOCs and other gases to classify patients with COPD, lung cancer, or other respiratory complications [19,20,21,22,23,24,25,26,27,28,29,30]. Binson et al. used XGBoost with an array of 5 Figaro VOC sensors to differentiate between a healthy control group and lung cancer group with an accuracy of up to 93% [20]. Liu et al. utilized a much wider array of 19 different gas sensors to classify lung cancer patients from healthy controls with over 94% accuracy using SGL-SVM [22]. Chen et al. evaluated multiple different machine learning algorithms to discriminate between a high-risk group (likely to develop lung diseases later in life), COPD group, and lung cancer group [25]. They were also able to identify differences between patients with different stages of lung cancer (Stage I–IV). Aulia et al. [28] and Kumi et al. [29] developed E-noses alongside machine learning to classify between COPD and healthy controls. Al Hadi et al. examined the use of CO_2_ sensors to differentiate between healthy controls and patients with GERD and asthma with an accuracy of up to 84% [30].

### 1.3. Initial Prototype & Research Gap

**Figure 1:**
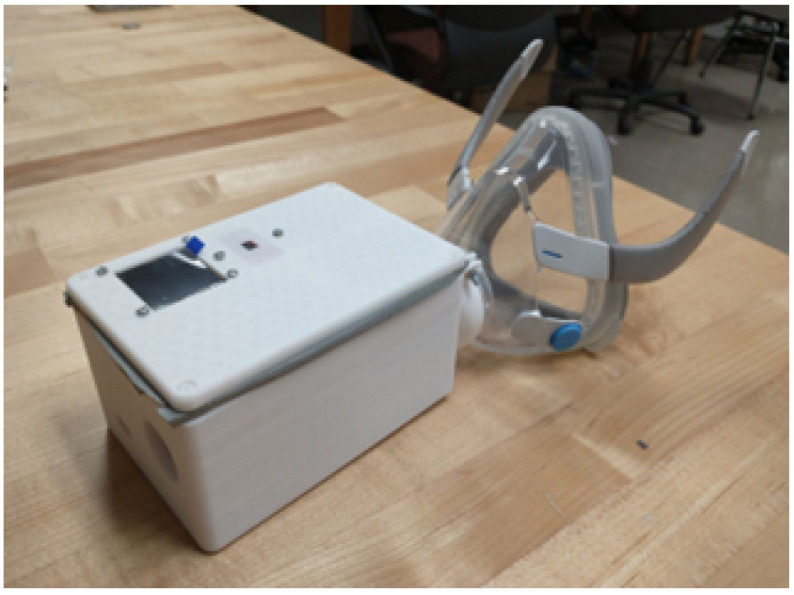
Initial prototype of our Pulmonary Assessment Mask (PAM).

Our initial prototype of a portable pulmonary assessment mask (PAM) is shown in Figure 1. The objective was to develop a proof-of-concept for a low-cost, portable respiratory monitoring and diagnostics system. The device could help in early at-home detection of COPD exacerbation events before symptoms manifest or worsen. The ability to evaluate respiratory health in the home is especially advantageous because of the reluctance of many individuals to seek health care, even when they know something is wrong or are experiencing symptoms [31]. There are many social determinants that affect willingness to seek medical help, but time commitment and financial concerns are the most common. By enabling low-cost at-home monitoring, respiratory healthcare can become more accessible.

Our initial prototype includes sensors to detect CO_2_, O_2_, temperature, and humidity. The PAM is also equipped with several metal oxide semiconductor (MOS) sensors to detect VOCs. Many E-Noses in the literature have employed MOS gas sensors due to their low cost and high sensitivity. However, they are somewhat limited by a number of drawbacks. Some MOS sensors suffer from reduced long-term stability, and may need to be re-calibrated, especially after storage for an extended period of time. MOS gas sensors also tend to have poor selectivity compared to other types of sensors [32] and react to a range of similar gases, though this disadvantage can be somewhat mitigated through the use of an array of multiple different sensors.

**Figure 2:**
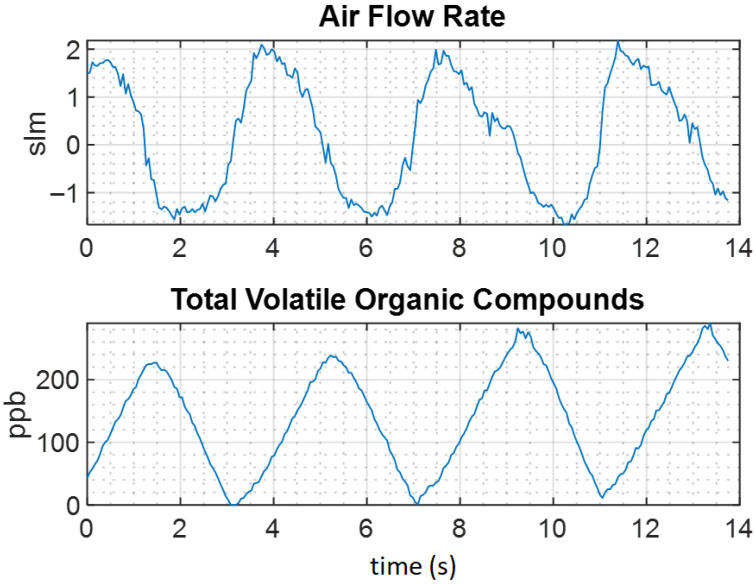
Example output of the PAM prototype showing failed convergence of VOC concentration to a steady-state value.

Upon initial testing of our prototype, we noted that some sensors (in particular, MOS sensors) are relatively slow to react, as shown in Figure 2. The long settling time associated with MOS gas sensors is the primary drawback we focus on in this paper. Adsorption-based sensors require a considerable period of time to reach steady-state compared to other types of sensors, which poses challenges in sampling human breath. The average human respiratory rate is typically between 12 and 20 breaths per minute [33], which is much shorter than the settling time of most MOS gas sensors.

In E-Nose papers that we reviewed, researchers overcome this limitation by collecting a sample from each patient in a Tedlar sampling bag (or similar gas storage medium) prior to testing [19,20,21,22,23,24,25,26,27]. Then, the exhaled breath sample is released into a sealed chamber, where the sensors are exposed to the sample until they reach their steady-state response.

**Figure 3:**
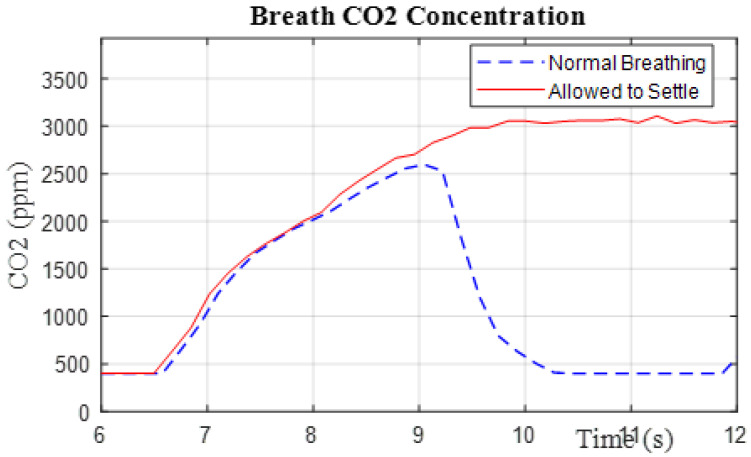
Comparison of the transient response versus the steady-state response of a gas sensor.

Our primary research thrust involves forecasting the steady-state value of a gas sensor using only a limited subset of data from the transient phase of the sensor’s response. This would allow for shorter sampling periods, possibly to the point where breath-by-breath analysis is achievable, without the need to store breath samples as an intermediate step. Figure 3 visually outlines the goal of this paper, where the objective is to compute the true final value of the sensor response (solid red curve at t=∞), using data from normal breathing cycles (dashed blue curve) where the sensor normally does not reach steady-state.

In this paper, we present two core contributions. We first present an accurate mathematical model that characterizes the typical response of a MOS gas sensor. Then, we present a novel forecasting methodology that enables the prediction of the final steady-state response of the sensor based on initial transient data.

This paper is organized as follows: Section 1 gives an overview of the motivation of this paper. Section 2 describes the basic principles of operation for MOS gas sensors. Section 3 covers the experimental setup and data analysis. Section 4 outlines the methodology we use to forecast the sensor response and the results we achieve. Finally, Section 5 briefly discusses the results and highlights the limitations of our work, as well as areas of future work we plan to pursue to improve on our current results.

## 2. Background

### 2.1. MOS Gas Sensor Operating Principles

**Figure 4:**
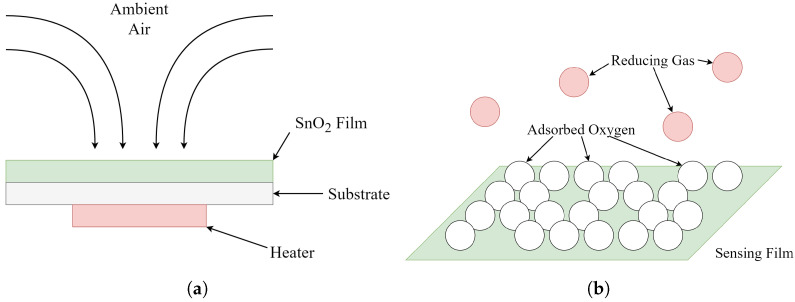
(**a**) Physical model of typical adsorptive metal oxide semiconductor gas sensors. (**b**) Sensor operating principle showing adsorbed oxygen molecules, and reducing gases causing desorption.

The structure of many MOS-type gas sensors is shown in Figure 4. A metal oxide sensing film (typically SnO_2_, but other oxides such as ZnO or WO_2_ are used as well) is deposited onto a substrate, where gas molecules are free to adhere to the surface of the film. A heating element is installed below the sensor, which raises the operating temperature of the device and allows for a faster sensor response than would otherwise be possible at room temperature. This configuration is commonly called a micro-hotplate sensor in the literature.

After being heated to operating temperature, oxygen molecules readily adsorb to the surface of the oxide film, binding the free electrons in the sensing film via a chemisorption reaction. This decreases the conductance of the sensor, resulting in a relatively high resistance when the sensor is in clean air.

In the presence of a reducing agent R (such as VOCs, CO, and other types of gases), the reaction in Equation (1) occurs, which results in the desorption of oxygen molecules, releasing free electrons back into the sensing film. This process increases the conductance of the film, which can be measured when attached to an external circuit.

(1) $$O^{-}+R\to RO+e^{-}$$

### 2.2. Langmuir Response Model

The sensor response can be represented simply using Langmuir adsorption kinetics, which models an ideal mono-layer adsorption process. If θ represents the fractional occupancy of sites on the adsorbent, then its derivative is intuitively given by the difference of the adsorption rate $r_{ad}$ and desorption rate $r_{d}$, given in Equation (2).

(2) $$\frac{d\theta (t)}{dt}=r_{ad}-r_{d}$$

In the Langmuir model, $r_{ad}$ and $r_{d}$ can be modeled as in Equation (3). The adsorption rate is proportional to the number of free sites, and the desorption rate is directly proportional to the number of molecules already adsorbed to the film.

(3) $$r_{ad}=k_{ad}\left[1-\theta \left(t\right)\right],\;r_{d}=k_{d}\theta \left(t\right)$$

Given Equations (2) and (3), we arrive at Equation (4). This is an ordinary differential equation with a first-order exponential function as its solution, shown in Equation (5). This can be further converted to a simple first-order exponential function, as the values of $k_{ad}$ and $k_{d}$ are not of interest to us for our application. Existing works outlined in Section 1.2 have demonstrated that raw sensor values can be directly used to make classification decisions.

(4) $$\frac{d\theta (t)}{dt}=k_{ad}\left[1-\theta \left(t\right)\right]-k_{d}\theta \left(t\right)$$

(5) $$\theta \left(t\right)=\frac{k_{ad}-C_{1}e^{-t(k_{ad}+k_{d})}}{k_{ad}+k_{d}}$$

### 2.3. Stretched Exponential Response Model

Although Langmuir adsorption is often suitable for modeling MOS gas sensors, it does not take into account more complex effects such as surface imperfections, multi-layer adsorption, and Knudsen diffusion of molecules into the porous surface of the oxide film.

Other studies [34,35] have found that adding a stretching exponent β to extend the Langmuir model to a stretched exponential function is a better approximation of the sensor response with complex non-ideal effects. A general form of the stretched exponential is shown in Equation (6).

(6) $$x_{\beta }\left(t\right)=ae^{-{\left(t/\tau \right)}^{\beta }}$$

## 3. Data Collection & Analysis

### 3.1. Data Collection

The data used in this paper was collected from three different gas sensors identified in Table 1. These low-cost sensors, most notably the first two sensors manufactured by Figaro USA (Rolling Meadows, IL, USA), are used in many E-Nose devices in existing works. They exhibit some cross-sensitivity, which is noted in Table 1. However, since their response characteristics are different, we include both sensors for consideration in this work.

It should be noted that the third sensor is an electrochemical sensor rather than a MOS sensor. We included this sensor to test the applicability of our methodology to other types of gas sensors as well. While the SGX-4OX sensor does have a faster response time than MOS-based sensors, the settling time of the sensor still exceeded 10 s during our observations. While it might be possible for a healthy individual to exhale for this long to obtain a reading, it could present challenges for individuals with COPD or other lung complications. Therefore, we also examine our forecasting methodology on this sensor. All of the sensors used in the system were sourced from Digikey Electronics.

**Figure 5:**
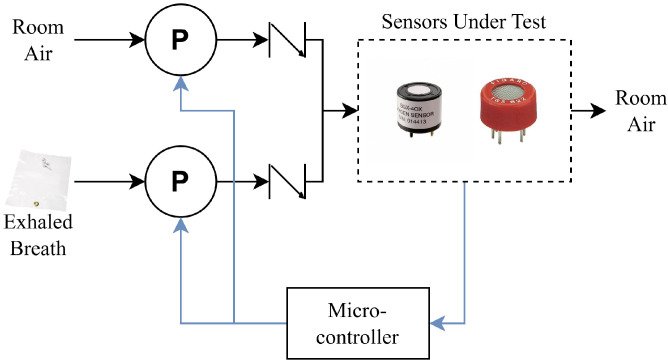
Block diagram of the data collection apparatus.

**Figure 6:**
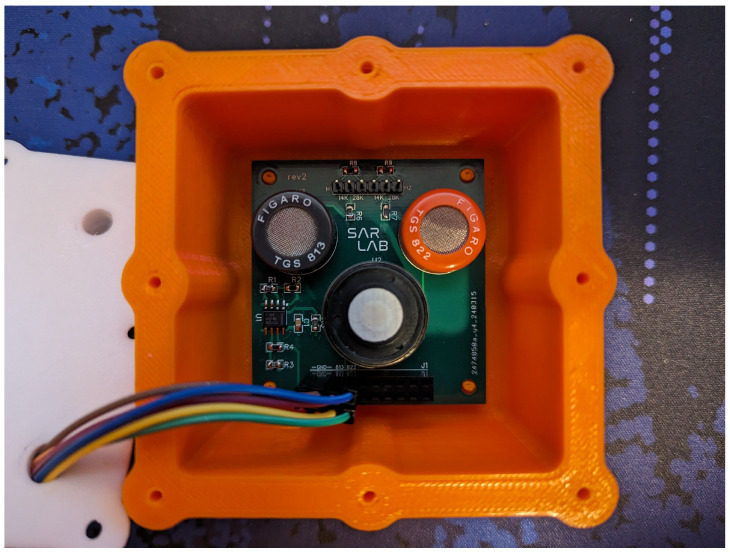
Gas sensors mounted inside the sensing chamber.

A block diagram of the experimental E-Nose test setup used in this paper is shown in Figure 5, where the arrows signify airflow. The sensors are shown inside their enclosure in Figure 6. The system utilizes two vacuum pumps that can pump either fresh air from the ambient environment, or exhaled breath samples that are collected in a sampling bag prior to testing. The system is capable of simulating a human breathing cycle by alternating the flow of external air and the exhaled breath sample, though in this paper, we let the sensors fully settle to their steady-state response in order to validate our forecasting methodology.

The resistance of the TGS 822 and TGS 813 sensors were recorded using a simple voltage divider circuit with the output connected to a 12-bit ADC. Both sensors were operated at the manufacturer-specified power rating for the microheater: 660 mW for the TGS 822, and 835 mW for the TGS 813. Both sensors underwent an initial burn-in time of continuous power-on for 7 days before being used, and a warm-up period of at least 10 min thereafter before each data collection run. The SGX-4OX, unlike the other two sensors, is an electrochemical sensor that outputs a small current of approximately 90 μA at 21% oxygen concentration (typical ambient air). This current was measured across a 100 Ω precision shunt with a 2-stage amplifier circuit to boost the signal by a gain of 225. All three sensors were sampled at a rate of 20 Hz during each experiment, though a lower sampling frequency could be used due to the comparatively slow response speed of the gas sensors.

Exhaled breath samples were collected in 10 L foil sampling bags prior to testing. The first step of the experimental procedure involved connecting the sampling bag to the E-Nose, where a vacuum pump was used to pump the contents of the sampling bag through the sensing chamber for 90 s, exposing the sensors to the exhaled breath sample (henceforth, the exposure period). After the exposure period, the sensors undergo a recovery period lasting for 4 min, where ambient room air is pumped through the sensing chamber. This ensures the system is purged of any trace gases from the exposure period, and that the gas sensors have ample time to return to their initial steady-state before the next experiment. This procedure was repeated 25 times by collecting data from one of the authors. For this paper, the system was tested at room temperature, and did not consider the effects of varying humidity or sensitivity of the sensors to different gas mixtures. We plan to address these in a follow-up work; we discuss this in more detail in Section 5.

After data collection, MATLAB R2025a was used to pre-process the data by converting the ADC values to engineering units. A median filter with a window size of 3 was also applied to suppress external transient noise and erroneous readings from each of the sensor responses. Since a temporal window spanning 3 samples (approximately 100 ms) is significantly lower than the response speed of the sensors used in the experiment, a window size of 3 preserves the integrity of the data as much as possible, while removing these erroneous sensor readings.

**Figure 7:**
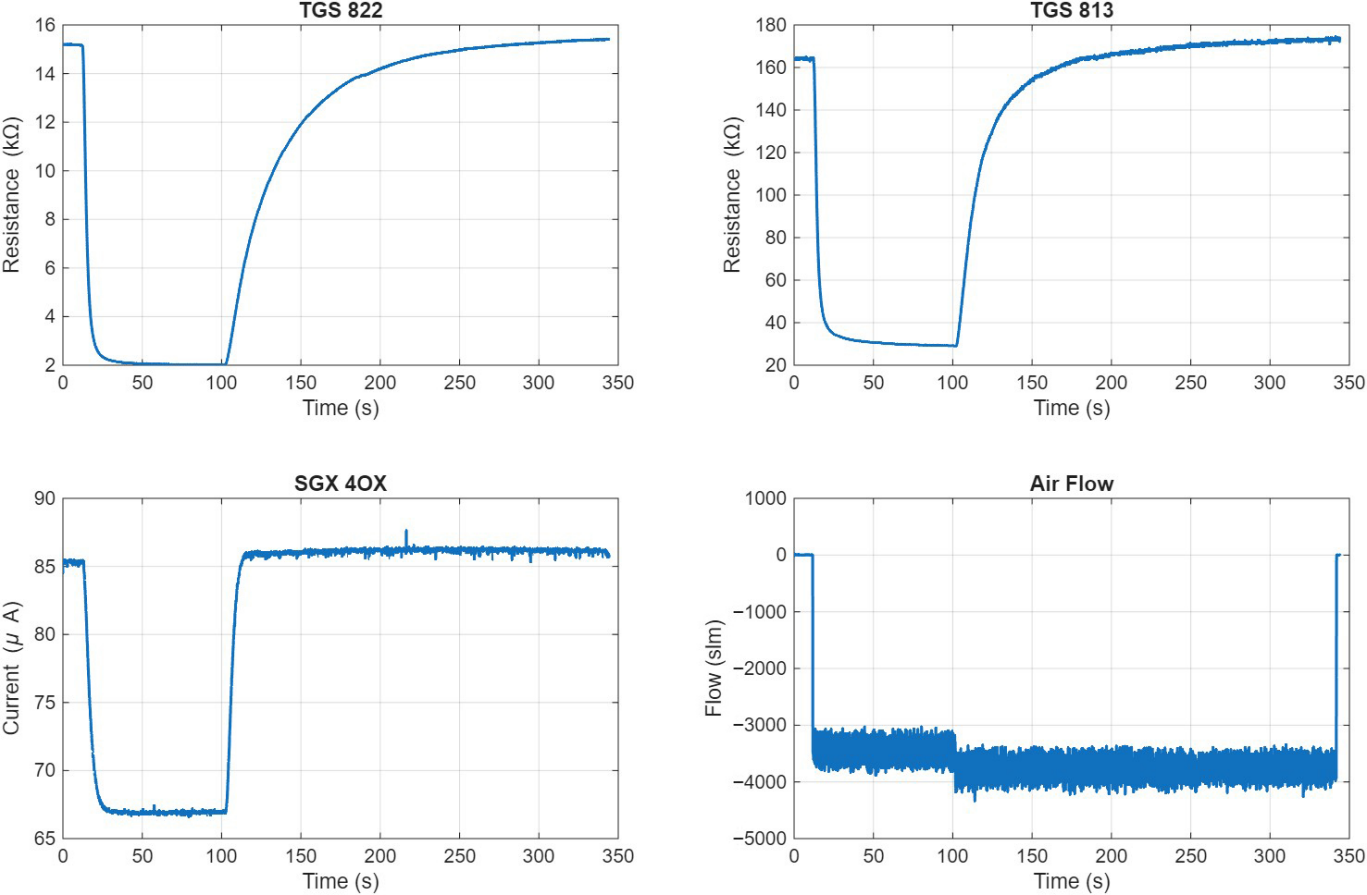
Example instance of data collected from the test setup, including the response of each sensor and the recorded airflow.

Figure 7 shows a sample of pre-processed data collected from the testing apparatus. A Sensirion SFM3200 airflow sensor was also included to measure the airflow through the sensing enclosure, which was used to determine the beginning of the exposure period. This was done by finding the time $t_{0}$ where the airflow exceeds 0.2 slm, indicating that the vacuum pump is enabled. The sensor response for the three gas sensors between $t_{0}$ and $t_{0}+85$ was then extracted. This yielded 25 samples of pre-processed data, each containing three sensor responses 85 s long.

### 3.2. Data Analysis & Model Fitting

MATLAB’s Curve Fitting Toolbox was used to find and fit an appropriate model to the collected gas sensor data. The fit was conducted using the nonlinear least squares (NLLSQ) method, and goodness-of-fit was evaluated using the root mean squared error (RMSE) between the ground-truth and model prediction across all sample points on the curve.

Four different mathematical models were evaluated based on the modeling assumptions outlined in Section 2. These are summarized in Table 2, where the average RMSE and normalized RMSE (normalized to the range of the signal) for each model across all sensor responses is shown. It should be noted that the fit to each sensor is shown separately, as the difference in response characteristics of the sensors makes a direct comparison meaningless.

**Figure 8:**
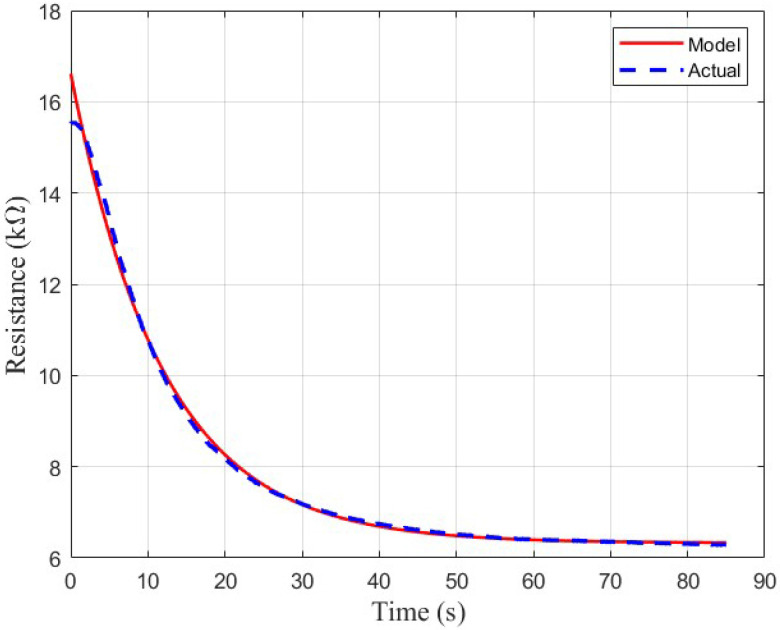
Qualitative fit of both first-order models to a typical TGS 822 response curve.

Two first-order exponential models of the forms $ae^{-t/\tau }+o$ and $ae^{-{\left(t/\tau \right)}^{\beta }}+o$ were fit first, as these most closely resemble the Langmuir dynamics we expected to observe. Although these two models characterize the general shape of the response well, they fail to model the initial transient behavior of the sensor due to their first-order nature. Both of these first-order models performed very similarly in most scenarios, as the NLLSQ solver found a solution where β≈1.0 for many of the breath samples, indicating that model 2 tends to degenerate towards model 1. This is reflected in the small difference in performance between models 1 and 2 in Table 2. The fit of both models 1 and 2 can be seen from a qualitative perspective in Figure 8.

We believe that the initial transient observed in the sensor response is due to second-order dynamics caused by the initial diffusion of gas into the sensing chamber. Thus, to improve the goodness-of-fit, two second-order models were considered. The first is a simple second-order exponential in the form of $a_{1}e^{-t/\tau_{1}}+a_{2}e^{-t/\tau_{2}}+o$, and the second model is a sum of one exponential function and a stretched exponential function, in the form of $a_{1}e^{-{\left(t/\tau_{1}\right)}^{\beta }}+a_{2}e^{-t/\tau_{2}}+o$.

**Figure 9:**
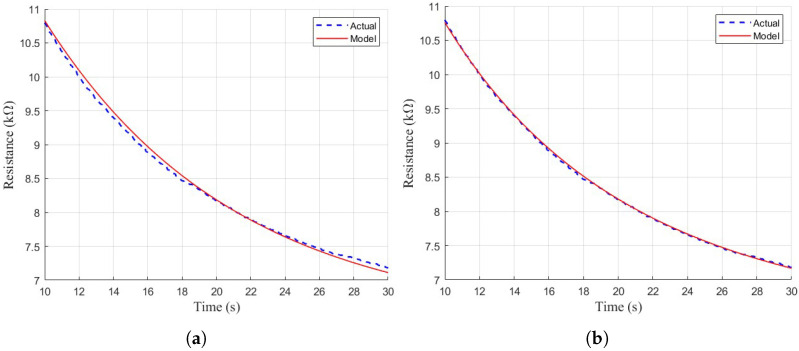
(**a**) The zoomed-in view of model 3’s fit on a typical TGS 822 response curve. (**b**) The zoomed-in view of model 4’s fit on a typical TGS 822 response curve.

The two second-order models exhibited a much better fit to the sensor data. Although the simple second-order model mostly fits the shape of the curve, there is some non-exponentiality to the sensor response where the model deviates slightly from the actual response, as shown in Figure 9. The stretched exponential function is better able to model these non-exponential characteristics, where the simple model slightly overshoots or undershoots in some areas compared to the ground-truth.

**Figure 10:**
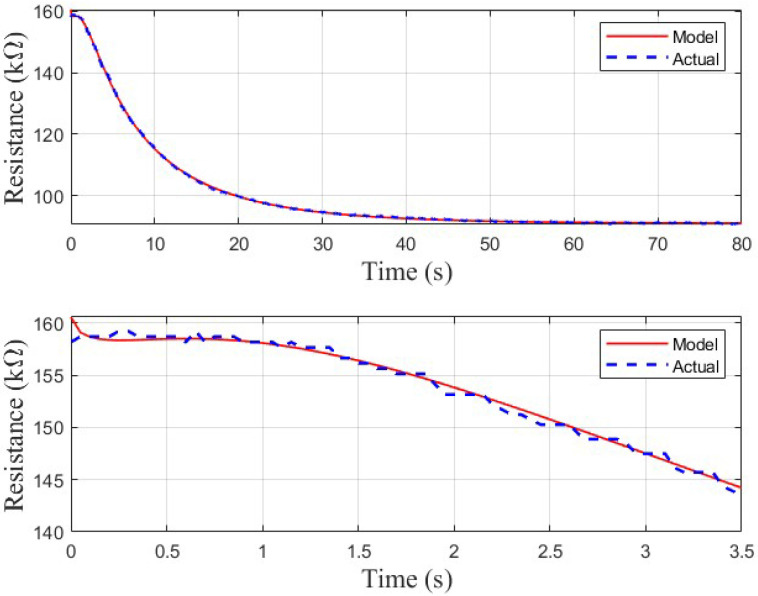
Qualitative analysis of the fit of the final chosen model to a TGS 813 response curve.

The second-order model including the stretched exponential is overall a much better fit, as shown in Figure 10. Aside from a slight transient at the beginning of the curve, the model fits the ground-truth sensor response significantly better than the simple second-order model. This is verified by Table 2, which indicates that the second-order stretched exponential model exhibits a lower RMSE on average than the simple second-order model for all sensors.

Aside from the models shown in Table 2, higher-order models of the general form shown in Equation (7) were also examined, with *n* being the order of the model. Third-order models and higher did not demonstrate improved performance compared to second-order models due to over-parameterization. Fitted models commonly contained two or more confounding exponential terms with wide confidence intervals, indicating that those terms are redundant and can freely change oppositely to each other with little impact on the resulting model.

(7) $$x\left(t\right)=a_{1}e^{-{\left(t/\tau_{1}\right)}^{\beta }}+\sum_{i=2}^{n}a_{i}e^{-t/\tau_{i}}+o$$

Lastly, the addition of a delay term *d* was also investigated, as shown in Equation (8). Similarly to the higher-order models that were examined, the addition of a delay to each exponential term also resulted in over-parameterization, which made convergence of the NLLSQ algorithm more difficult to achieve.

(8) $$x\left(t\right)=a_{1}e^{-{\left(\left(t+d\right)/\tau_{1}\right)}^{\beta }}+\ldots$$

Thus, the final model we selected was the summation of a stretched exponential term and one additional standard exponential, shown by Equation (9) (Model 4 from Table 2). We found that this model most consistently models the behavior of each gas sensor in our testing apparatus.

(9) $$x\left(t\right)=a_{1}e^{-{\left(t/\tau_{1}\right)}^{\beta }}+a_{2}e^{-t/\tau_{2}}+o$$

## 4. Sensor Forecasting Methodology & Results

Once we determined that Equation (9) was an accurate mathematical model for each of the sensors under test, we examined three different methods to forecast the final value $t_{\infty }$ of the sensor response given only part of the response curve from $t_{0}$ to $t_{n}$, where data after $t_{n}$ was not provided to the model to train on. For all 3 methods, $t_{n}$ was chosen at 10 s, though a few additional values for $t_{n}$ were also examined in Section 4.1. Section 4.1 outlines our initial attempts at using NLLSQ to forecast the final sensor value. Section 4.2 outlines the results we achieve using 2 different deep neural networks, which saw greater success than attempting to use NLLSQ.

### 4.1. Nonlinear Least Squares

Our initial attempt at forecasting involved examining the possibility of using the NLLSQ method. This was accomplished by fitting a model on data from $t_{0}$ to $t_{n}$ of the response, and then comparing the resulting model to the ground-truth sensor response. Table 3 shows the RMSE of models generated by using the first 85 s (full response), 40 s, 20 s, and finally 10 s of the response. RMSE was computed for the full sensor response curve, and was averaged across all samples for each sensor and each value of $t_{n}$.

**Figure 11:**
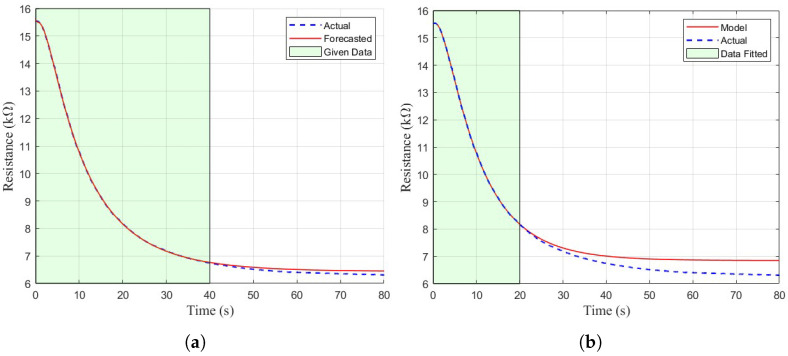
(**a**) Fit of the model to the TGS 822 using the initial 40 s of data. (**b**) Fit of the model to the TGS 822 using the initial 20 s of data.

It can be observed from Table 3 that the quality of the fitted model is significantly degraded when less data is used to generate the model. Figure 11 shows a qualitative view of the model fit with only the initial 40 s and 20 s of data used in the nonlinear least squares algorithm, respectively. In both cases the model fits well to the given data, but the extrapolative capability of NLLSQ clearly degrades with lower amounts of given data. In these cases, the final value of the response deviates significantly from actuality and flattens out much earlier or much later than in reality, as shown in Figure 11. This result was expected, as a simple curve-fitting algorithm does not have the capability to forecast or predict future data based on only a small subset of initial data points.

### 4.2. Neural Networks

To improve upon our ability to forecast the final value of the sensor response, we examined the use of neural networks to predict the final value of the sensor response. We tried two different approaches, which are outlined Section 4.2.1 and Section 4.2.2.

For both NN approaches, we elected to augment the dataset with artificial data derived from the real data samples, as only 25 samples were collected in Section 3. Before augmentation, we split the dataset using a 80–20 train–test split, resulting in 20 samples being used for training and 5 for testing. After this step, augmentation was done by copying samples from the original dataset and then scaling the amplitude and DC offset of the sensor response curve by a random value. Since these augmented samples are derived from real data samples, we recognized that overfitting could occur due to hidden features in the data being replicated inadvertently across multiple training samples. To address this concern and mitigate the probability of overfitting the data, a small amount of AWGN was also added to each signal to suppress these hidden features in the data. After augmentation, our dataset consists of 125 samples in total. Since we conducted the train-test split before augmentation, this results in a stratified sampling that ensures both the training and testing set contain a proportional amount of real and generated samples.

#### 4.2.1. Prediction of Mathematical Parameters

**Figure 12:**
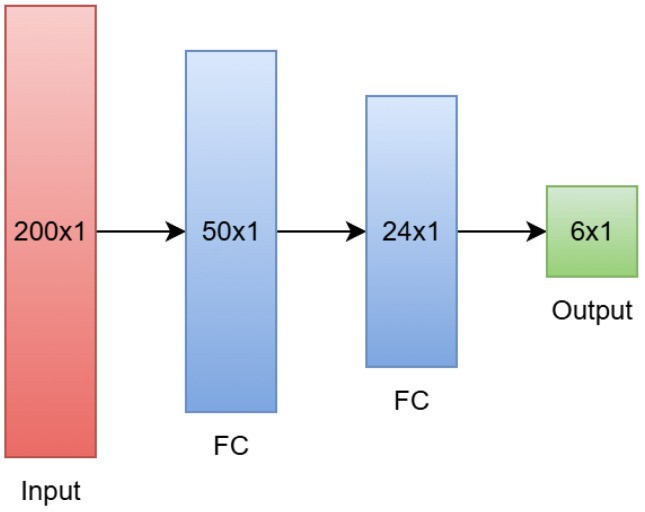
Architecture of the parameter-based neural network.

The first NN architecture we used attempts to predict the mathematical parameters of the initial model chosen in Section 3.2. The overall architecture is shown in Figure 12. The first 200 samples of the sensor response (corresponding to 10 s of data) are used as inputs to the NN, which pass through two fully-connected hidden layers of size 50 and 24. A leaky ReLU activation function is applied after each fully-connected layer. After the second layer, an output layer of size 6 gives the predictions of each of the six coefficients for the model outlined in Equation (9).

During training, data scaling was also conducted to improve the convergence of the model during training, as the large disparity in magnitude between some predictors and targets led to poor stability during both training and inference. All predictors were divided by a scaling factor $F_{i}$, defined in Equation (10), where $x_{i,0}$ is the sensor response at $t_{0}$ for the $i^{th}$ training instance. Similarly, all targets and outputs were scaled by $F_{o}$, a vector where each element $F_{o,j}$ is defined in Equation (11).

(10) $$F_{i}=\max\left\{x_{i,0}:i=1\to n\right\}$$

(11) $$F_{o,j}=\max\left\{y_{i,j}:i=1\to n\right\};\;j=1\to 6$$

Training was conducted over 100 epochs using the Adam optimizer, with an initial learning rate of 2 × 10^−4^ and a minibatch size of 16. Table 4 shows the training loss of the model with 5-fold cross-validation. For each sensor, there is some variability in the loss across folds, which suggests that some training instances are more difficult to learn than others. This is likely due to the small size of the dataset, which we discuss in Section 5.

#### 4.2.2. Direct Forecasting of Final Value

The second NN architecture we used is mostly identical to the one in Section 4.2.1. However, instead of predicting the mathematical coefficients of Equation (9), we train this model to predict the final value of the sensor response directly. As such, the architecture is identical to the one shown in Figure 12, with the exception of the output layer, which simply has one output. Aside from this difference, the training process is identical to what is outlined above in Section 4.2.1. Table 5 shows the training loss of the model, which shows the same general trends as Table 4 regarding the quality of the model’s training.

### 4.3. Forecasting Results

The results of each of the three methods for each sensor is shown in Table 6 and Table 7 for final value prediction of the response of each sensor. Table 6 displays the mean absolute error (MAE) of the final value prediction (of $t_{\infty }$) between the ground-truth and estimated value, with the mean absolute percentage error (MAPE) in parenthesis next to it.

**Figure 13:**
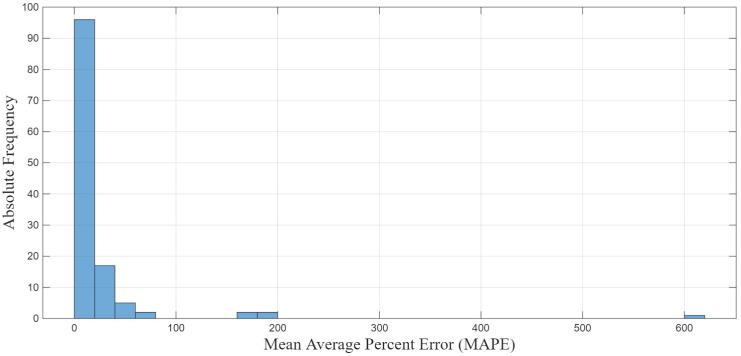
Histogram of MAPE values showing few instances of extreme outliers, indicating hard scenarios for the model.

Upon further analysis of the individual error values for each inference, we noted that most inferences during testing exhibited a much lower error than the MAE metrics displayed in Table 6. These high MAE values were caused by the dominance of few test set samples where the final value prediction was poor, which is demonstrated in Figure 13. Upon further analysis of the offending test set samples, we noted that one of the data samples exhibited an anomalous response profile compared to the rest of the data, which we believe to be the cause of this extreme outlier in the prediction. Table 7 highlights this by displaying the geometric mean absolute error (GMAE) and geometric mean absolute percentage error (GMAPE); the geometric mean reduces the impact of extreme outliers compared to the arithmetic mean.

While the failure of these models to provide a high-quality prediction for these outliers is still of concern, the performance of the model is otherwise encouraging for most of the samples in the test set. Both NN models outperform a naive attempt at final value forecasting using NLLSQ by a significant margin.

Discounting some extreme outliers, both DNN models predict the final value within approximately 8% and 4.5% for the TGS 822 and TGS 813 sensors, and the final-value based DNN model predicted the final output value of the SGX-4OX sensor within about 1% on average. It should be noted, however, that due to the faster settling time of the SGX-4OX sensor, the results are less meaningful than the other two sensors. Given the current results in Table 6 and Table 7, it is difficult to determine if there is a meaningful difference between using a NN to predict the parameters of a mathematical model versus directly predicting the output. In some situations, these models lie within 1% MAPE of each other in performance, which is well within the noise floor caused by variability in model weight initialization and training.

## 5. Conclusions & Future Work

In this paper, we found a suitable second-order model for MOS-type gas sensors that closely resembles the observed behavior of the gas sensors used during the experiment. This was indicated by the model prediction’s low RMSE when compared to the collected ground-truth data. Our initial attempts at forecasting the future response of the sensor used this model with the nonlinear least squares method. However, we found that a naive approach such as NLLSQ is insufficient for determining the future response of the sensor. Since the algorithm optimizes to find the best possible fit for the data it is given, it is clear that there is not enough information in the initial transient response of these gas sensors to correctly predict the steady-state value using NLLSQ. The use of NNs improved the results significantly compared to NLLSQ, though some challenges still present themselves in the results obtained from these methods.

One of the primary limitations of this work is the small size of the dataset used to train and test the forecasting models. Only 25 samples were collected during the data acquisition phase, and while this was later augmented to 125 total samples based on real sensor responses, the size of the dataset still presents challenges in reliably training forecasting models to achieve accurate predictions of the final sensor value. In a future work, we plan to conduct a follow-up study to significantly expand the size of the collected dataset to alleviate this problem. By having a larger dataset, we will be able to better generalize the model to different situations and be able to better account for differences in sensor responses between individuals.

In addition to increasing the size of the dataset, we also plan to examine the applicability of this method to real-time breath-by-breath analysis in a future work. Currently, we mimic the approach of other works in the literature by collecting breath samples and allowing the gas sensors to remain exposed to the sample until they reach the steady-state. However, if we vary the airflow to mimic a human breathing cycle, as well as use this traditional approach of letting the sensors settle to obtain ground-truth sensor responses, then we can extend this framework to operate on cyclical breathing data as well. The primary challenge that accompanies breath-by-breath analysis is the fact that the recovery period between breaths is a few seconds at most during a normal breathing cycle, which is not sufficient for a MOS sensor to return to its baseline value. However, there is still a “cleaning” process that must be done in between individual trials, or when swapping from one individual to another. During this time, the sensors would be allowed to return to their baseline value. By doing this, the hope is to be able to perform the prediction on the user’s first breath into the mask, bypassing the need for an exposure period entirely.

Another challenge that arises from breath-by-breath analysis is the effect of airflow on the sensors’ time constant (s). In this paper, we assume a constant airflow into the sensing enclosure. However, for a human breathing cycle, this assumption does not hold true. In Section 3.1, we establish that our data collection apparatus has the capability to measure airflow, as well as the capability of varying the airflow into the sensing enclosure if desired. In a follow-up work, we aim to incorporate the airflow data into the model to determine how airflow affects the sensor response. This would then enable us to simulate a human breathing cycle to demonstrate real-time breath-by-breath analysis capabilities.

Lastly, we plan to implement temperature and humidity sensors to our testing enclosure, as well as to test different gas mixtures, in future works. It is known that both humidity and temperature have an impact on the sensitivity of MOS sensors. Most MOS gas sensors also exhibit cross-sensitivity with multiple different gases, with the scale of the sensor response varying depending on the gases in contact with the sensing film. In future works, we plan to integrate these variables into our models to improve the prediction accuracy across breath samples of varying humidity, temperature, and composition.

## Figures and Tables

**Table 1:** List of gas sensors used in the experimental setup.

Vendor	Sensor	Gas Selectivity	Type
Figaro	TGS 822	Methane, CO, Ethanol, Acetone, Benzene	MOS
Figaro	TGS 813	Methane, CO, Ethanol, Propane, Hydrogen	MOS
SGX SensorTech (Neuchâtel, Switzerland)	SGX-4OX	Oxygen	Electro-chemical

**Table 2:** Evaluation of the RMSE * and normalized RMSE (NRMSE, in parenthesis) of different sensor models compared to the ground-truth.

Model	TGS 822	TGS 813	SGX-4OX
(1) $ae^{-t/\tau }+o$	0.1740 (0.0155)	1.9344 (0.0176)	0.5722 (0.0300)
(2) $ae^{-{\left(t/\tau \right)}^{\beta }}+o$	0.1705 (0.0151)	1.7903 (0.0156)	0.5722 (0.0300)
(3) $a_{1}e^{-t/\tau_{1}}+a_{2}e^{-t/\tau_{2}}+o$	0.0932 (0.0086)	1.6849 (0.0143)	0.1089 (0.0057)
(4) $a_{1}e^{-{\left(t/\tau_{1}\right)}^{\beta }}+a_{2}e^{-t/\tau_{2}}+o$	**0.0459 (0.0042)**	**1.1519 (0.0097)**	**0.0905 (0.0048)**

* RMSE and NRMSE is computed between all data points on the ground-truth and estimated response, and is averaged across all collected samples. Note: Numbers in bold indicate the best model for each sensor.

**Table 3:** Evaluation of the RMSE * and normalized RMSE (NRMSE, in parenthesis) compared to the ground-truth given different fitting durations.

$t_{n}$ (Fitted Data)	TGS 822	TGS 813	SGX-4OX
85 s	0.0459 (0.0042)	1.1519 (0.0097)	0.0905 (0.0048)
40 s	0.0937 (0.0097)	1.7119 (0.0161)	0.0965 (0.0051)
20 s	0.2183 (0.0234)	2.9024 (0.0289)	0.1198 (0.0062)
10 s	2.1531 (0.1888)	6.6649 (0.0864)	0.1471 (0.0078)

* RMSE is computed between all data points on the ground-truth and estimated response, and is averaged across all collected samples.

**Table 4:** Training loss of the parameter-based NN over 5 folds.

Sensor	Fold 1	Fold 2	Fold 3	Fold 4	Fold 5
TGS 822	0.0677	0.0851	0.1019	0.0742	0.0947
TGS 813	0.0893	0.1086	0.1349	0.0776	0.0936
SGX-4OX	0.1614	0.1455	0.1505	0.1426	0.1486

**Table 5:** Training loss of the final-value-based NN over 5 folds.

Sensor	Fold 1	Fold 2	Fold 3	Fold 4	Fold 5
TGS 822	0.8090	0.5013	0.3994	0.5063	0.7346
TGS 813	6.3426	4.7328	4.5560	4.2473	2.3518
SGX-4OX	1.0199	0.4795	1.2061	0.9311	0.9374

**Table 6:** Mean average error (MAE) and mean average percentage error (MAPE, in parenthesis) of the final value prediction using three different models.

Method	TGS 822	TGS 813	SGX-4OX
NLLSQ	3.8599 (95.7%)	12.6880 (33.0%)	16.4407 (24.4%)
DNN (Param)	**0.4930 (18.3%)**	3.7313 (13.48%)	2.1913 (3.3%)
DNN (Final Value)	**0.4732 (17.8%)**	**3.6598 (9.7%)**	**0.8895 (1.3%)**

Note: Numbers in bold indicate the best method for each sensor.

**Table 7:** Geometric mean average error (GMAE) and geometric mean average percentage error (GMAPE, in parenthesis) of the final value prediction using three different models.

Method	TGS 822	TGS 813	SGX-4OX
NLLSQ	1.9097 (58.3%)	10.0291 (22.0%)	6.1089 (9.1%)
DNN (Param)	**0.2676 (8.2%)**	**2.0732 (4.6%)**	1.4520 (2.2%)
DNN (Final Value)	**0.2522 (7.7%)**	**1.9936 (4.4%)**	**0.5960 (0.9%)**

Note: Numbers in bold indicate the best method for each sensor.

## Data Availability

The data collected and used in the study is openly available on GitHub at https://github.com/SAR-Research-Lab/data_adsorptive_gas_sensor_forecasting (accessed on 15 January 2026).
